# Mechanical Dentistry

**Published:** 1862-10

**Authors:** B. W. Franklin

**Affiliations:** New York City


### MECHANICAL DENTISTRY.

[Read before the American Dental Convention at Trenton Falls, Aug. 6, 1862.

BY B. W. FRANKLIN, OF NEW YORK CITY.

Mr. President:—The propriety of inserting temporary dentures having been questioned by many good dentists, it would be well for us as a profession, if possible, to come to some understanding whereby a more uniform practice would obtain on this as well as other important operations connected with our practice.

That much has been said against inserting dentures upon the gums immediately after the teeth have been extracted, I am aware, and much more could be said against the practice, when common gutta percha, silver, or even thin gold plates are employed as a base.

All yielding substances have the effect to depress or materially change the position and character of the natural gum at the point of yielding in the base. Silver being a soft, yielding metal, if there were no other objection to its use, as a base for artificial dentures, this alone should condemn it forever for this purpose.

We have seen the alveolar ridge not only very considerably distorted by the use of silver plates, but have also seen the antagonizing teeth forced out of their true positions by reason of articulating against artificial teeth upon silver, or other yielding substances.

The force exerted in mastication with artificial as well as the natural upper teeth is upward and outward, and in case the base upon which the teeth are mounted loses its power of reaction, the force of mastication is exerted in a direction to produce the greatest possible injury to the anterior alveolar borders.

The base of the teeth resting upon that portion of the plate which covers the anterior portion of the natural ridge, it must be obvious, if any changes in the position of this portion of the plate take place, the force being mainly exerted inward against the anterior alveolar border, would produce absorption of the alveoli, and a doubling over or folding in of the soft parts.

These results, and they are frequent, have doubtless led to the prejudice against the insertion of temporary dentures, and I am frank to admit if such results were the unavoidable consequences of wearing temporary dentures, it, of itself, would be sufficient reason for their abandonment.

But fortunately for our profession and mankind, the introduction of continuous gum, and later still the vulcanite, has in a great measure established confidence, and will very speedily entirely revolutionize the views of members of our profession in regard to the utility of inserting temporary dentures I am satisfied that the more rigid and unchangeable the base, the less disfigurement of the natural ridge.

Some dentists attach so much importance to the presence of teeth in the mouth, that they actually make the temporary denture before extracting the natural teeth. This in theory is very beautiful, but whether in practice it is always feasible, I will not pretend to say; but no time, in my judgment, should be lost after the teeth are extracted before substituting artificial ones.

The advantage gained by the employment of proper temporary sets will more than compensate for any and all objections that can be truthfully urged against their use.

The greatest pains should always be taken in the adaptation of the plates, position, size, shade and expression of the teeth, and every part of the denture should be as faithfully and perfectly made, as though it was designed for permanent use.

When a temporary set is adapted in every respect and possesses all the requirements that the case demands, the patient will much more readily acquire the use of the permanent one. This of itself is no small advantage gained.

The bungling, unskillful manner in which many dentists put up their temporary work can not be too severely condemned. Every mechanical contrivance intended for use in the mouth should be made with especial reference to its resting upon living sensitive parts, and that these parts are often extremely susceptible to the slightest irritating cause, and when irritated in some cases, the most serious results follow.

Impressions for temporary dentures, as for all others, should be taken in thin plaster. The indentations in the model filled up before forming the plate, thus producing a series of chambers on the ridge of the plate, which ultimately become filled up with healthy granulations, preventing the absorption of the ridge, and the consequent changes that would go on in the absence of the protection which the plate affords.

When the soft gums are exposed to the pressure of masticating upon them, absorption is promoted and great changes in the general expression of the individual is a consequence.

It is universally acknowledged that the more the gums shrink away, the greater the change in the contour of the face, the expression, and all the features, making up the individuality of the patient's being. We all have observed that those who have lost their teeth, and have been longest without them, have undergone the greatest transformation in the external configuration of their faces, and it is but reasonable that it should be so. When the gums are unprotected by teeth, they have to sustain the entire force of mastication. The constant use and pressure on them promotes absorption and great recession of the soft parts, and hence the changes so noticeable in most persons who have lost their teeth and before their replacement by artificial substitutes.

If, on the contrary, the gum is covered with a plate made from some of the unyielding substances, the palatine surface of the mouth sustains most of the force exerted in mastication.

If the teeth have a proper position and the plate is firmly retained in its place after the ridge is slightly absorbed, the palatine arch then sustains the whole of the force; now if the plate is sufficiently rigid so as not to allow that portion of it covering the natural ridge to spring upon the gum, the gum will in a large majority of cases conform to the original shape of the plate, thus preserving nearly its original thickness, and preventing in a great degree the deformity consequent upon the absorption of the ridge.

Another important advantage gained by inserting temporary dentures is, the preservation of the natural intonation of the voice, and when the permanent set is inserted, the patient, being accustomed to the first set, will readily become so to the permanent ones.

I desire here to mention one important point to be observed in the construction of temporary or permanent dentures. It is the proper articulation or bearings of the upper with the lower teeth. The greatest pressure in all cases should be a little posterior to the center of the denture; this is not only material to the utility of the dentures in masticating food, but essential to the preservation of the natural ridge, and also the expression of the face as well as the voice.

In a large majority of cases, when an upper denture is inserted in a mouth having but the front inferior teeth remaining, the pressure against the upper denture being so far anterior to the center, causes an absorption of the anterior portion of the natural ridge, which in a short time will become a softened pendulous mass.

To obviate this unnatural state of things, our patients should in all cases be made to understand that a partial under set is absolutely necessary to be inserted in order to realize a reasonable benefit from the use of an upper denture. I am aware that the insertion of partial under sets heretofore has been an operation not over satisfactory to the patient or to the dentist. The difficulties in being able to manipulate metallic plates to these cases has led to a pretty general abandonment of the practice.

Continuous gum work from the pliability of platinum was doing much to restore confidence in the utility of partial under dentures, but owing to the lack of confidence by some in its strength and the difficulties experienced by others, and the total inability of a much larger number to acquire the art of working this beautiful and simple process, the public and the profession have derived but little practical benefits comparatively from its use for partial under sets.

It seems that it is a part of the great plan of Providence to reserve for us till these latter days the best things in his gift, and on a little reflection it is but natural that this should be the case. As we progress, we are brought into a condition the better to appreciate and apply for our own and others' use things that in our undeveloped state we could not appreciate at all, and hence vulcanite has been reserved for us till this time, and it is a most gratifying fact that the profession and the public are now enjoying the blessings of science in developing, and art in adapting, this most wonderful substance to the wants of mankind in more than a thousand different ways, none of which are of more importance than its use for temporary plates, and for partial under dentures.

The most useful things in nature are always the most simple in their operations and application to the wants of man. It is their very simplicity that makes them adapted to the wants of man in his imperfectly developed state.

Let us, then, as a profession, employ the instrumentalities within our reach, and strive to use as much as we need in an earnest, honorable effort to serve mankind, remembering that our noble profession will be advanced or retarded by our own individual efforts.

